# Functional exhaustion of antiviral lymphocytes in COVID-19 patients

**DOI:** 10.1038/s41423-020-0402-2

**Published:** 2020-03-19

**Authors:** Meijuan Zheng, Yong Gao, Gang Wang, Guobin Song, Siyu Liu, Dandan Sun, Yuanhong Xu, Zhigang Tian

**Affiliations:** 1 Department of Clinical Laboratory, First Affiliated Hospital of Anhui Medical University, Hefei, Anhui China; 2 Department of Clinical Laboratory, Fuyang Second People’s Hospital, Fuyang Infectious Disease Clinical College, Anhui Medical University, Fuyang, Anhui China; 3 Division of Molecular Medicine, Hefei National Laboratory for Physical Sciences at Microscale, the CAS Key Laboratory of Innate Immunity and Chronic Disease, School of Life Sciences, University of Science and Technology of China, Hefei, Anhui China; 4 Institute of Immunology, University of Science and Technology of China, Hefei, Anhui China

**Keywords:** Immunology, Cell biology

In December 2019, a novel coronavirus was first reported in Wuhan, China.^1^ It was named by the World Health Organization as severe acute respiratory syndrome coronavirus 2 (SARS-CoV-2) and is responsible for coronavirus disease 2019 (COVID-19). Up to 28 February 2020, 79,394 cases have been confirmed according to China’s National Health Commission. Outside China, the virus has spread rapidly to over 36 countries and territories.

Cytotoxic lymphocytes such as cytotoxic T lymphocytes (CTLs) and natural killer (NK) cells are necessary for the control of viral infection, and the functional exhaustion of cytotoxic lymphocytes is correlated with disease progression.^2^ However, whether the cytotoxic lymphocytes in patients infected with SARS-CoV-2 become functionally exhausted has not been reported.

We showed that the total number of NK and CD8^+^ T cells was decreased markedly in patients with SARS-CoV-2 infection. The function of NK and CD8^+^ T cells was exhausted with the increased expression of NKG2A in COVID-19 patients. Importantly, in patients convalescing after therapy, the number of NK and CD8^+^ T cells was restored with reduced expression of NKG2A. These results suggest that the functional exhaustion of cytotoxic lymphocytes is associated with SRAS-CoV-2 infection. Hence, SARS-CoV-2 infection may break down antiviral immunity at an early stage.

SARS-CoV-2 has been identified as a genus β-coronavirus, and it shares 79.5% sequence homology with SARS-CoV.^3^ In our cohort of 68 COVID-19 patients admitted to The First Affiliated Hospital (Hefei) and Fuyang Hospital (Fuyang), both of which are part of Anhui Medical University in China, there were 55 cases of mild disease (MD) and 13 cases of severe disease (SD). Patients were aged 11–84 years, and the median age of patients was 47.13 years. The percentage of male patients was 52.94%. Consistent with previous studies, many patients had fever (80.88%), cough (73.53%), and sputum (32.36%) upon admission. The prevalence of other symptoms (e.g., headache, diarrhea) was relatively low (Supplementary Table 1). The clinical features of patients infected with SARS-CoV-2 was consistent with those reported by Chen and colleagues.^4^

Upon admission, the neutrophil count was remarkably higher in SD patients than in MD cases, whereas the lymphocyte count was significantly lower in SD cases than in MD cases. The concentration of total bilirubin, D-dimer, and lactate dehydrogenase in blood was higher in SD patients than that in MD patients. Levels of alanine aminotransferase and aspartate aminotransferase were slightly higher in SD cases than those in MD cases. Levels of albumin and hemoglobin were lower in SD patients than those in MD patients (Supplementary Table 2). Specifically, T cell and CD8^+^ T cell counts were decreased significantly in MD and SD patients compared with those in healthy controls (HCs). The number of T cells and CD8^+^ T cells was significantly lower in SD patients than that in MD cases. The counts of NK cells were reduced remarkably in SD patients compared with those in MD cases and HCs (Fig. 1a).

**Fig. 1:**
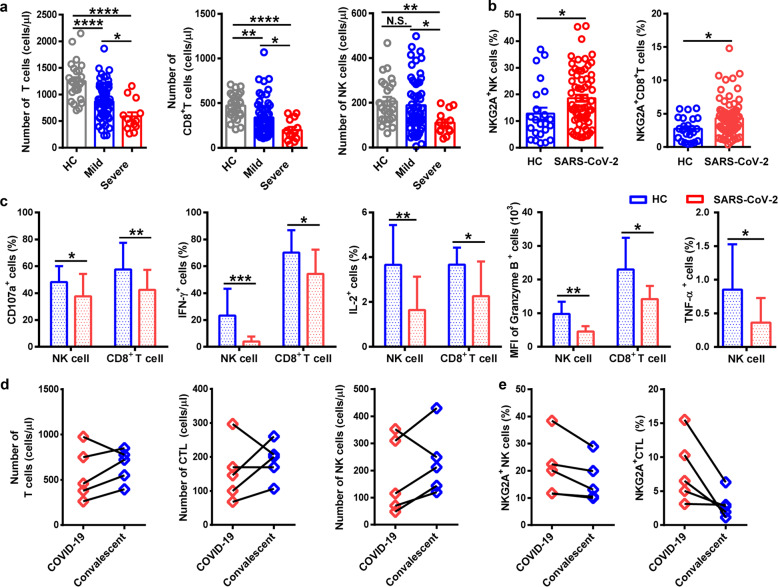
NKG2A^+^ cytotoxic lymphocytes are functionally exhausted in COVID-19 patients. **a** Absolute number of T cells, CD8^+^ T cells, and NK cells in the peripheral blood of healthy controls (*n* = 25) and patients with mild (*n* = 55) and severe (*n* = 13) infection with SARS-CoV-2. **b** Percentages of NKG2A^+^ NK cells and NKG2A^+^CD8^+^ T cells in the peripheral blood of healthy controls (*n* = 25) and patients infected with SARS-CoV-2 (*n* = 68). **c** Expression of intracellular CD107a, IFN-γ, IL-2, and granzyme-B in gated NK cells and CD8^+^ T cells and percentage of TNF-α^+^ NK cells in the peripheral blood of patients infected with SARS-CoV-2 and healthy controls. **d** Total number of T cells, CTLs, and NK cells in the peripheral blood of COVID-19 patients and convalescing patients. **e** Percentages of NKG2A^+^ NK cells and NKG2A^+^ CTL in the peripheral blood of COVID-19 patients and convalescing patients. Data are mean ± SEM. Unpaired/paired two-tailed Student’s *t* tests were conducted. *p* < 0.05 was considered significant. *p < 0.05, **p < 0.01, ***p < 0.001, ****p < 0.0001; N.S., not significant

As an inhibitory receptor, NKG2A has been demonstrated to induce NK cell exhaustion in chronic viral infections.^5^ Notably, NKG2A expression on NK and CD8^+^ T cells results in functional exhaustion of NK and CD8^+^ T cells.^6^ In patients infected with SARS-CoV-2, NKG2A expression was increased significantly on NK and CD8^+^ T cells compared with that in HCs (Fig. 1b). Next, to identify the role of NKG2A on the function of NK and CD8^+^ T cells, levels of CD107a, interferon (IFN)-γ, interleukin (IL)-2, granzyme B, and tumor necrosis factor (TNF)-α were measured through staining of intracellular cytokines. We found lower percentages of CD107a^+^ NK, IFN-γ^+^ NK, IL-2^+^ NK, and TNF-α^+^ NK cells and mean fluorescence intensity (MFI) of granzyme B^+^ NK cells in COVID-19 patients than those in HCs. Consistent with these findings, COVID-19 patients also showed decreased percentages of CD107a^+^ CD8^+^, IFN-γ^+^CD8^+^, and IL-2^+^CD8^+^ T cells and MFI of granzyme B^+^CD8^+^ T cells, compared with those in HCs (Fig. 1c). Taken together, these results suggest the functional exhaustion of cytotoxic lymphocytes in COVID-19 patients. Hence, SARS-CoV-2 may break down antiviral immunity at an early stage.

In our setting, ~94.12% of patients were administered antiviral therapy (Kaletra®). Chloroquine phosphate was used in 7.35% of patients, and the proportion of patients treated with IFN was 64.71%. In addition, 48.53% patients received antibiotic treatment (Supplementary Table 3). Comparison of the total number of cytotoxic lymphocytes (including CTLs and NK cells) after therapy was carried out. The total number of T cells and NK cells recovered in the convalescent period in four of the five patients, and the total count of CTLs was restored in the convalescent period in three of the five patients (Fig. 1d). Hence, efficacious therapy was accompanied by an increased number of T cells, CTLs, and NK cells. Importantly, the percentage of NKG2A^+^ NK cells was decreased in the convalescent period compared with that before treatment among five patients. Similarly, five patients showed a decreased percentage of NKG2A^+^ CTLs in the convalescent period (Fig. 1e). These findings suggest that downregulation of NKG2A expression may correlate with disease control in COVID-19 patients.

We showed that NKG2A expression was upregulated on NK cells and CTLs in COVID-19 patients with a reduced ability to produce CD107a, IFN-γ, IL-2, granzyme B, and TNF-α. Also, the percentage of NKG2A^+^ cytotoxic lymphocytes was decreased in recovered patients infected with SARS-CoV-2, which strongly suggests that NKG2A expression may be correlated with functional exhaustion of cytotoxic lymphocytes and disease progression in the early stage of COVID-19. Although exhaustion of T and NK cells occurs in human chronic infection and tumorigenesis, T cell apoptosis (which is regarded as the host mechanism involved in chronic infection and cancer) also occurs in SARS-CoV infection.^7^ Thus exhausted NKG2A^+^ cytotoxic lymphocytes may be present in COVID-19 patients. With regard to our finding that the percentage of NKG2A^+^ cytotoxic lymphocytes was decreased after antiviral therapy in COVID-19 patients, efficacious control of SARS-CoV-2 infection is related to reduce expression of NKG2A on cytotoxic lymphocytes. Therefore, in COVID-19 patients with severe pulmonary inflammation, SARS-CoV-2-induced NKG2A expression may be correlated with functional exhaustion of cytotoxic lymphocytes at the early stage, which may result in disease progression. Moreover, immune inhibitory “checkpoint” receptors that result in exhaustion of NK and T cells have been demonstrated in chronic infection and cancer. Importantly, checkpoint inhibitors such as anti-PD-1 and anti-TIGIT help to reinvigorate exhausted responses from T or NK cells in the context of chronic infection and cancer.^8,9^ NKG2A is thought to be a novel inhibitory molecule on immune-checkpoint blockade.^10^ Taken together, these data highlight the importance of improving the immune response of NK cells and CTLs and avoiding exhaustion of cytotoxic lymphocytes at the early stage of SARS-CoV-2 infection. Therefore, targeting NKG2A may prevent the functional exhaustion of cytotoxic lymphocytes and consequently contribute to virus elimination in the early stage of SRAS-CoV-2 infection.

## Supplementary information

Supplementary Materials
