# Supplementary material for: Functional exhaustion of antiviral lymphocytes in COVID-19 patients
Source: Cell Mol Immunol. 2020 Mar 19;17(5):533–5. doi: 10.1038/s41423-020-0402-2 (PMC7091858; doi:10.1038/s41423-020-0402-2)
Supplement: Supplementary file 1 — Supplementary Materials [file 41423_2020_402_MOESM1_ESM.docx]

**Supplementary Materials**

**Methods**

**Sample collection**

Peripheral-blood samples of patients infected with SARS-CoV-2 and healthy controls were collected from The First Affiliated Hospital of Anhui Medical University (Hefei, China) and Fuyang Hospital of Anhui Medical University (Fuyang, China). All patients were diagnosed with SARS-CoV-2 infection. According to clinical features, we divided them into the “severe disease” group and “mild disease” group. Detailed patient information is summarized in the Supplementary Table. Approval of the study protocol was obtained from the Ethics Committee of The First Affiliated Hospital of Anhui Medical University

**Flow cytometry**

Suspensions of lymphocytes isolated from fresh blood were stained with mouse anti-human monoclonal antibodies (Extend Data Table). A BD FACSCanto flow cytometer (Becton Dickinson) was used to assess stained cells and data were analyzed using FlowJo VX (TreeStar). Intracellular staining of cytokines was done after suspensions of lymphocytes were stimulated with medium containing 10% fetal bovine serum (Gibco) with 30 ng/ml phorbol 12-myristate 13-acetate (Sigma–Aldrich), 1 μg/ml ionomycin (Merck Millipore) or 2.5 μg/ml monensin (Sigma–Aldrich) for 4 h. Then, cells were fixed and permeabilized and stained with the antibodies of intracellular cytokines. The antibodies used for flow cytometry are also shown in this study: FITC–anti-CD4, PE–anti-Granzyme B, PE-anti–TNF-α, PE–anti-IL-2, PerCP-CY5.5-anti–IFN-γ, PE-CY7–anti-CD107a, APC-H7–anti-CD8, BV421–anti-CD56, BV605–anti-CD3, Multitest 6-colour TBNK (all from BD Biosciences), PE-CY7–anti-NKG2A (Beckman).

**Statistical analyses**

Data are the mean ± SEM and were analyzed using Prism 5.0 (GraphPad). The independent samples *t*-test was used to evaluate quantitative variables. P < 0.05 was considered significant. *p < 0.05, **p < 0.01, ***p < 0.001, ****p < 0.0001; N.S., not significant.

**sTable 1 Characteristics of patients infected with SARS-CoV-2**

|  | All patients  （n=68） | Mild  （n=55） | Severe  （n=13） |
| --- | --- | --- | --- |
| Age  Sex ·  Man  Woman  Fever  Cough  Sputum  Headache  Diarrhoea  Wuhan exposure | 47.13(11-84)  36/68(52.94%)  32/68(47.06%)  55/68(80.88%)  50/68(73.53%)  22/68(32.36%)  8/68(11.76%)  3/68(4.41%)  12/68(17.65%) | 43.93(11-84)  26/55(47.27%)  29/55(52.73%)  42/55(76.36%)  41/55(74.55%)  16/55(29.09%)  8/55(14.55%)  2/55(3.64%)  9/55(16.36%) | 60.69(59-82)  10/13(76.92%)  3/13(23.08%)  13/13(100%)  9/13(69.23%)  6/13(46.15%)  0/13(0%)  1/13(7.69%)  3/13(20.08%) |

**sTable 2 Laboratory findings of patients infected with SARS-CoV-2 on admission to hospita**

|  | All patients  (n=68) | | Mild  (n=55) | | Severe  (n=13) | P value | |
| --- | --- | --- | --- | --- | --- | --- | --- |
| White blood cell count, × 10⁹/L  Neutrophil count, × 10⁹/L  Lymphocyte count, × 10⁹/L  Monocyte count , × 10⁹/L  CD4^+^ T, Cell/μl  B cell , Cell/μl  Haemoglobin, g/L  Platelet count, × 10⁹/L  Prothrombin time, s  D-dimer, mg/L  Albumin,g/L  Alanine aminotransferase, U/L  Aspartate aminotransferase, U/L  Total bilirubin, mmol/L  Potassium, mmol/L  Sodium, mmol/L  Creatinine, μmol/L  Lactate dehydrogenase, U/L  C-reactive protein,mg/L | 5.981(2.83-14.24)  3.806(1.24-12.07)  1.37(0.32-2.33)  0.432(0.24-0.74)  491.391(108.47-1040)  157.451(39-356)  129.389(87-170)  238.3(79-380)  11.713(9.7-17.7)  1.181(0.12-8.71)  37.573(9.7-47.4)  30.129(7-167)  24.176(10-89)  14.927(4.7-47.8)  4.003(2.69-5.17)  140.687(132.1-187)  69.209(39-209)  223.1(117-551)  28.819(0.1-343.8) | 5.744(2.83-14.24)  3.462(1.24-7.78)  1.487(0.57-2.33)  0.4309(0.24-0.68)  509.8(108.47-1040)  158.4(66-356)  131.8(87-170)  238.3(90-366)  11.58(9.7-14.9)  0.5207(0.12-3.48)  39.26(9.7-47.4)  24.7(7-67)  20.16(10-48)  13.62(4.7-47.8)  4.039(2.95-4.96)  141(132.9-187)  69.29(44-209)  186.9(117-303)  22.72(0.1-343.8) | | 6.868(3.07-13.26)  5.098(1.72-12.07)  0.9333(0.32-1.76)  0.4358(0.24-0.74)  413.3(140-885.63)  114.6(39-270.71)  120.3(101-162)  238.3(79-380)  12.12(10.4-17.7)  3.312(0.51-8.71)  32.65(26-38.6)  46(14-167)  35.92(15-89)  18.86(9.3-32.5)  3.894(2.69-5.17)  139.6(132.1-146.2)  63.62(39-106)  326.2.1(142-551)  48.35(0.3-156.9) | | | 0.1519  0.0111  <0.0001  0.9138  0.3145  0.1119  0.0426  0.9994  0.2390  <0.0001  0.0012  0.0090  0.0005  0.0337  0.4247  0.6008  0.5301  <0.0001  0.2515 |

**sTable 3 Treatments of patients infected with SARS-CoV-2**

|  | All patients  （n=68） | | Mild  （n=55） | | Severe  （n=13） | |
| --- | --- | --- | --- | --- | --- | --- |
| Kaletra  Hydroxychloroquine/ Chloroquine Phosphate  Interferon therapy  Antibiotic therapy | | 64/68(94.12%)  5/68(7.35%)  44/68(64.71%)  33/68(48.53%) | | 52/55(94.55%)  4/55(7.27%)  34/55(60.00%)  22/55(40.00%) | | 12/13(92.31%)  1/13(7.69%)  10/13(76.92%)  11/13(84.62%) |
